# Convolutional Neural Networks for Semantic Segmentation as a Tool for Multiclass Face Analysis in Thermal Infrared

**DOI:** 10.1007/s10921-020-00740-y

**Published:** 2021-01-03

**Authors:** David Müller, Andreas Ehlen, Bernd Valeske

**Affiliations:** 1 Fraunhofer Institute for Non-Destructive Testing IZFP, Campus E3 1, 66123 Saarbrücken, Germany; 2 Saarland University of Applied Sciences, htw saar, Goebenstr. 40, 66117 Saarbrücken, Germany

**Keywords:** Artificial intelligence, Machine learning, Thermography, Intelligent sensors, Health monitoring

## Abstract

Convolutional neural networks were used for multiclass segmentation in thermal infrared face analysis. The principle is based on existing image-to-image translation approaches, where each pixel in an image is assigned to a class label. We show that established networks architectures can be trained for the task of multiclass face analysis in thermal infrared. Created class annotations consisted of pixel-accurate locations of different face classes. Subsequently, the trained network can segment an acquired unknown infrared face image into the defined classes. Furthermore, face classification in live image acquisition is shown, in order to be able to display the relative temperature in real-time from the learned areas. This allows a pixel-accurate temperature face analysis e.g. for infection detection like Covid-19. At the same time our approach offers the advantage of concentrating on the relevant areas of the face. Areas of the face irrelevant for the relative temperature calculation or accessories such as glasses, masks and jewelry are not considered. A custom database was created to train the network. The results were quantitatively evaluated with the intersection over union (IoU) metric. The methodology shown can be transferred to similar problems for more quantitative thermography tasks like in materials characterization or quality control in production.

## Introduction

The ability to distinguish faces is natural and essential for humans. Through our rapid biological analysis, we can determine in fractions of a second whether a person is well-intentioned, make an assessment of their mood, or evaluate their emotional state. Face recognition is most commonly associated with applications in the optical field. In recent years, facial analysis has become an active research area in the field of machine vision. In the framework of security technology, facial recognition is a popular feature that is used on the computer as well as on the smartphone and in other areas. In social networks, it is used to tag faces on photos and to link images with the corresponding person. With extracted features, neural networks are also trained to make accurate predictions about age classification, gender, and more. Further fields of application are emotion research, but can also be used to provide conclusions about human diseases. Common and popular data sets on which networks can be trained for the aforementioned tasks in the optical area are e.g. 300-W [1] with 68 landmark annotations and bounding box initialization. Faces in the UTK dataset vary in pose and expression. The age range of the persons in the dataset varies from 0 to the age of 116 [2]. Also noteworthy is the Wider Facial Landmarks in-the-wild (WFLW) dataset with 10,000 faces each with 98 fully manual annotated landmarks [3]. The labeling of the data is a very time-consuming process as the data is usually labelled manually in the first step. RGB images are often used in the data sets, but face analysis in the optical field can also be done with gray images. Various studies have already tried to transfer existing approaches from the optical spectrum to the infrared spectrum. For example, in 2008, Hizem et al. tried to extract facial contours in the near infrared range (NIR) using classical edge detection methods [4]. Kopaczka et al. have worked with facial landmark detection and face tracking in long wave infrared (LWIR) images. For this purpose, they created an annotated database of thermal face image with 68 facial landmarks. Their face tracking method was then based on an active appearance model (AAM) [5]. The same author shows in [6] that a number of introduced algorithms for face detection in the visual spectrum can be trained to work in the thermal spectrum. In [7] they describe the used manual annotated database and provide an extension for emotion labels for LWIR images. Keong et al. describe a multi-spectral facial landmark detection approach based on a modified version of U-Net. Both face boundary and 68 landmark points could be trained simultaneously [8]. Our approach differs from the existing methods in that a segmentation approach was chosen for pixel precise detection. Image segmentation is the process of analyzing each individual pixel to identify whether it belongs to a defined class. Therefore, semantic segmentation is the process of combining homogeneous pixels into a defined group. The aim is to divide the image into subareas. It is therefore a matter of separating the image objects from the background and separating the image classes from each other. Due to the predicted result image, we can now examine the found class in more detail by overlaying it over the original image data. In the biomedical field, segmentation can be used for the detection of tumors, tissue damage, or for segmentation of computer tomography images [9]. In the automotive sector a common task is vehicle, lane and traffic shield detection, and segmentation. Classical methods are edge detection via e.g. Canny or Sobel filtering. A further classical method is the Hough transform for recognizing geometric figures such as circles, ellipses, and straight lines. However, by using artificial neural networks for this task, the probability of failure could be significantly reduced and the process of object recognition could be accelerated considerably. Face segmentation means to divide a face into several categorical classes which can then be addressed individually. The separation of a face into individual subareas enables an area-related and pixel accurate analysis. Especially in the context of the Covid-19 pandemic, the approach could be used as an alternative detection method for temperature extraction in relevant areas of the face (e.g. fever detection in the forehead area). Other approaches work with ROI for automatic face recognition, but calculate the average value over a total area and thus achieve an inaccurate value for further temperature analysis. Using the landmark point methods, only areas where landmarks are defined can be analyzed. In order to determine further points later, the position of the new coordinate would have to be calculated for each analysis in a complex way. The neural approach for infrared analysis presented in this work is based on the image to image translation problem. The trained network works both with single images and in video sequence analysis. The evaluation takes place with appropriate computer hardware in the microsecond range, therefore a live image analysis is also provided. Defined subareas can be detected with high precision and the reference to the face surface temperature can be made.

## Method

The process of segmental face recognition can be divided in several main phases:

- The creation of a training database with infrared images.
- Manual creation of multiclass segmentation mask annotations for different participants and varying face and head poses.
- Identification and development of a suitable neural network architecture for training.
- Network training and evaluation.
- Test the architecture with a dataset that is unknown to the network.

**Fig. 1:**
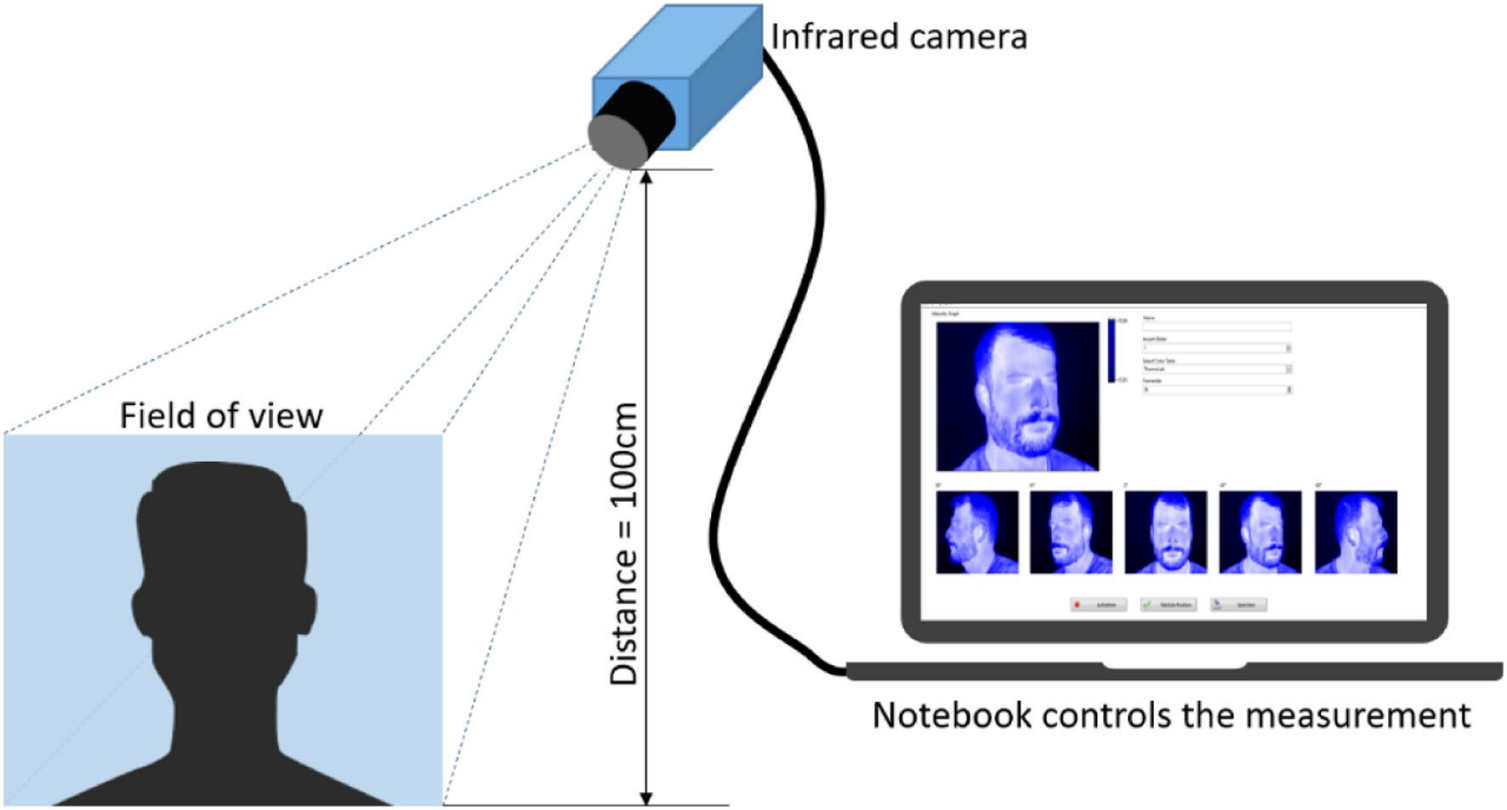
Measurement setup, consisting of an infrared camera and a computer. The participants were positioned at a distance of 1 m from the camera

In the first step, the infrared image database was created consisting of 240 different infrared images. For the data generation, a total of 4 persons were considered: 3 male and 1 female adult participant. Each participant was given a data protection declaration in advance, thus ensuring that the data could also be used in the context of a publication. It was also agreed that the data would only be used for the research activities and training of the network. The measurement setup is shown schematically in Fig. 1.

**Fig. 2:**
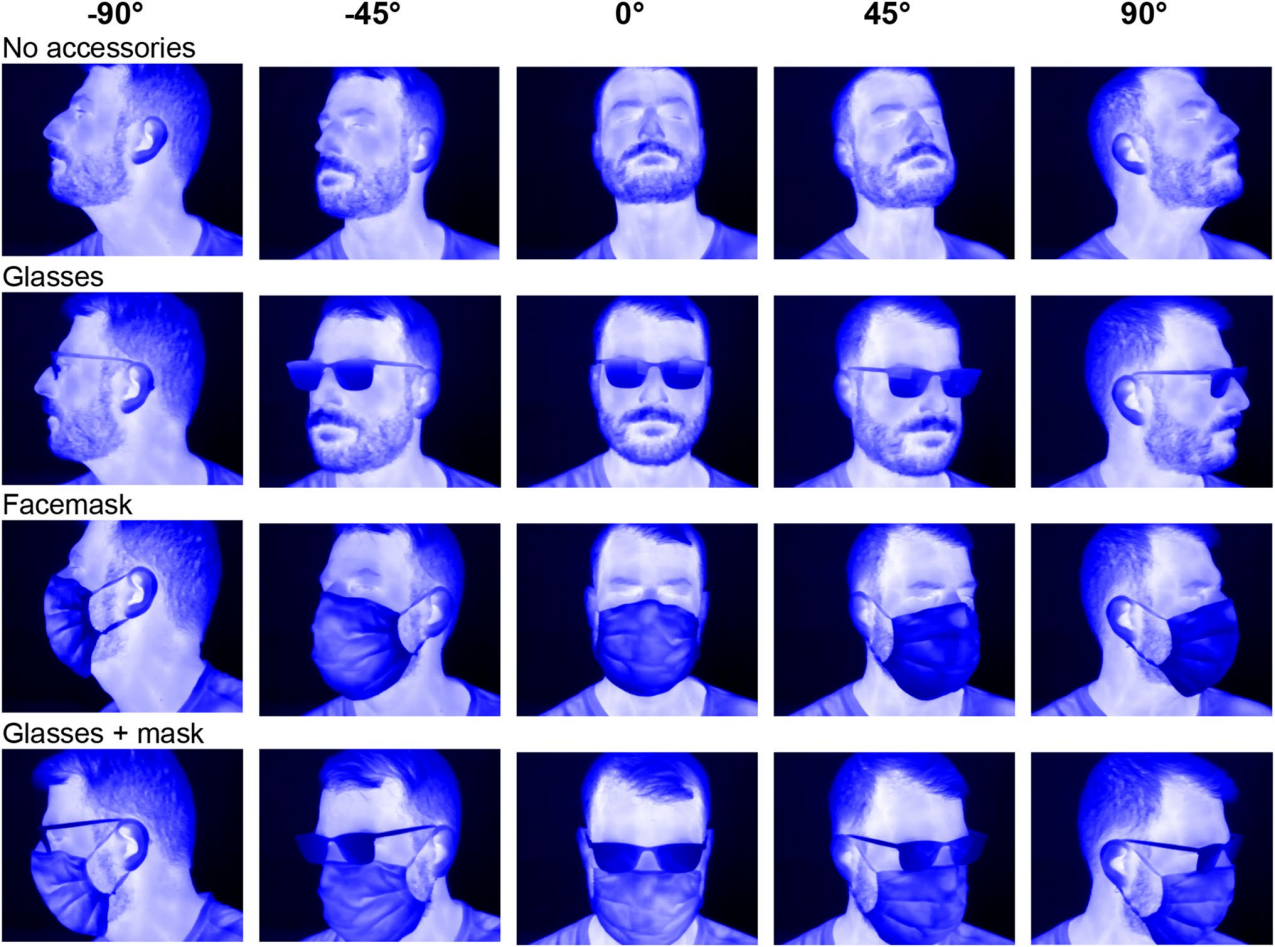
A selection of infrared images of one participant with varying head poses. Shown are the different camera angles from -90° to 90° horizontal and vertical head tilt down and up. Also shown are images with face accessories like glasses and a face mask

The infrared images were acquired with a FLIR AX5 bolometer camera. The AX5 operates at a frequency of 60 Hz, a resolution of 320 × 256 pixels, with a thermal sensitivity/noise equivalent temperature difference (NETD) of 50 mK at 30 °C at the long-wave infrared range (LWIR) of 7.5–13 µm. The manufacturer’s default calibration of the camera was used. The emissivity was set to nearly 1. This value corresponds well to the emissivity of human skin. The measurements took place in standard equipped office rooms. The participants were seated on a chair for the measurements. The camera was placed 1 m from the participants. Precautions were taken so that only one person at a time was visible to the camera. Furthermore, it was ensured that no disturbing influences, such as other heat sources, influenced the recording. In order to be able to obtain the best possible coverage of a participant's face and to achieve the best possible detection rate afterwards, different IR images of the participants with varying head position, facial expressions, and camera angles were acquired. Therefore, a database of 4 different sub datasets were created from each participant. These datasets included images without facial accessories as well as images with accessories like glasses and face masks. Each sub dataset consisted of 15 different recordings with varying cameras angles. Thus the head movement from − 90° to + 90° in 45° steps horizontally and with inclination of the head up and down was covered. Infrared image data from one participant are shown in Fig. 2. The training database for one participant, consisted of 60 different IR images. In total, a database of 240 infrared images was created.

**Fig. 3:**
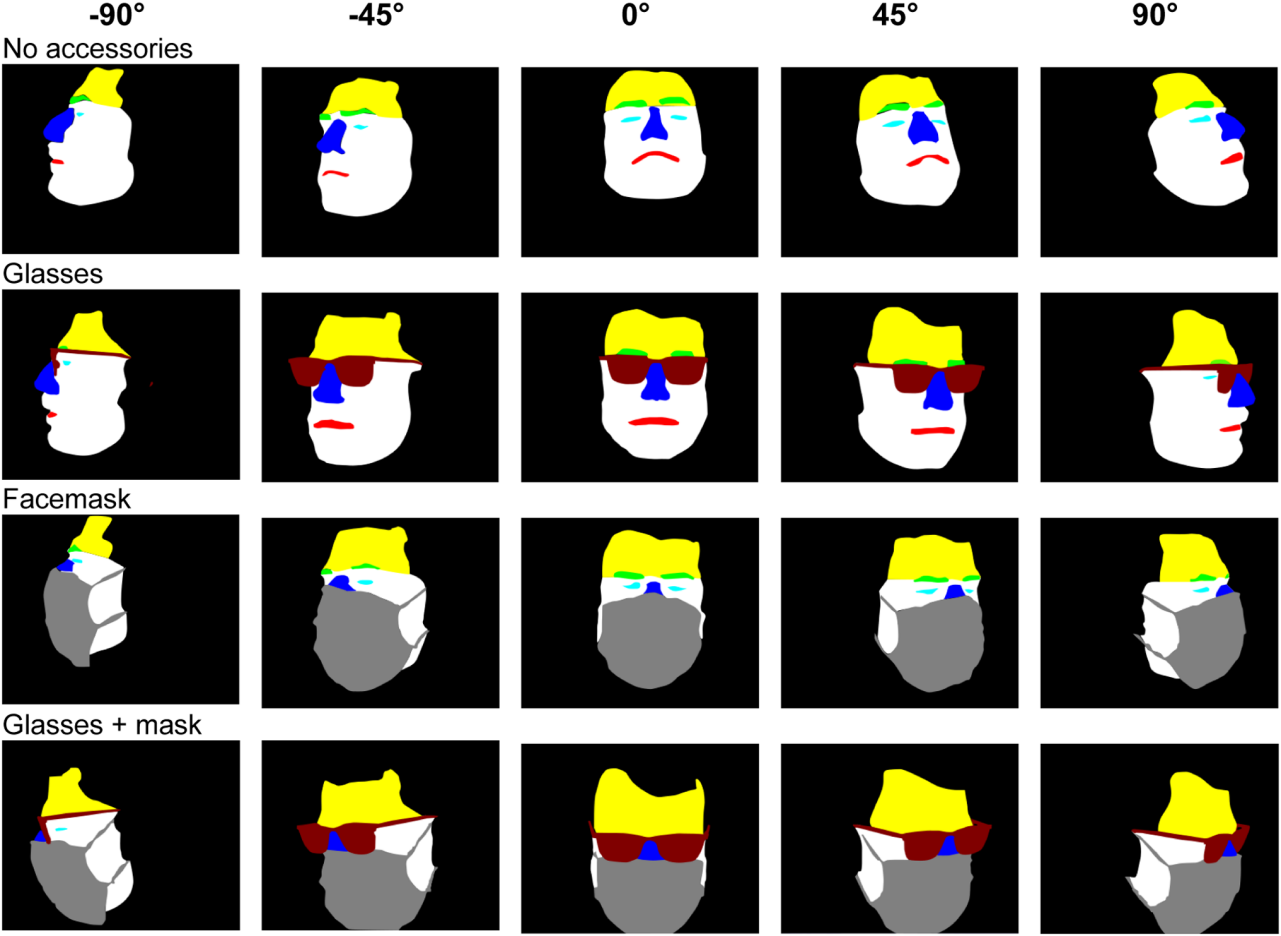
Facial segmentation multiclass masks for the defined camera angles, with and without facial accessories. The corresponding IR image is shown in Fig. 2

After recording the measurement data, a corresponding multiclass segmentation mask was created. All datasets were labeled manually. The manual labelling procedure is a very time-consuming process. The segmentation masks were created using a vector graphic software. The following facial areas were defined for the segmentation: eyes, eyebrows, nose, mouth, forehead, lower half of face, glasses, face mask, and background. In total, each multiclass segmentation image created contains 9 different classes. The definition of the individual classes was chosen according to natural tendency for important classes. Some of the segmentation masks are shown with reference to the Fig. 2 IR images in Fig. 3.

After the acquisition of the training data and creation of the corresponding multiclass segmentation masks database, the neural network can be designed and the data trained accordingly.

## Neural Network Architecture

**Fig. 4:**
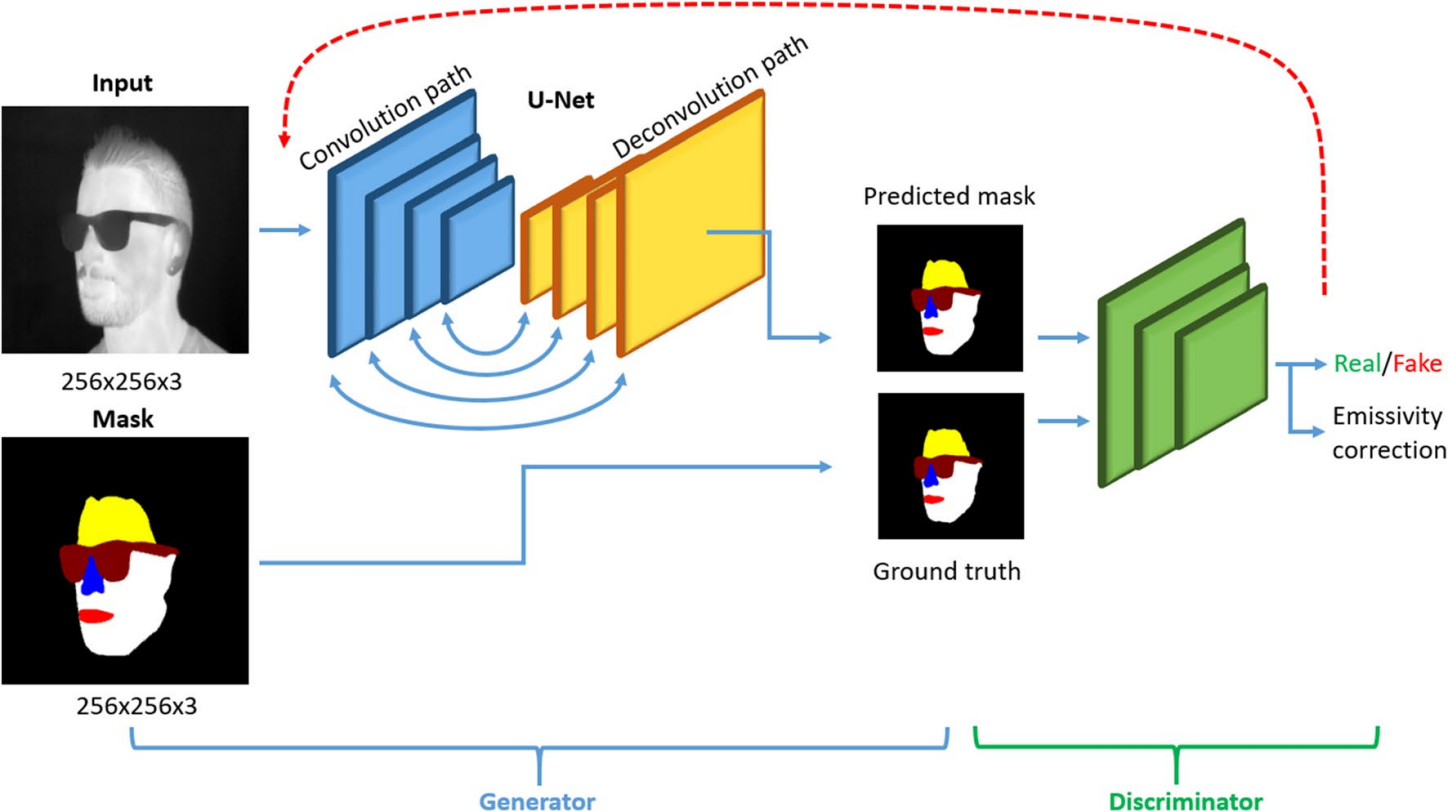
Schematic representation of the image to image translation architecture. Blue: generator path realized by a U-Net, Green: Discriminator path realized by a CNN which distinguishes between the real/fake data and gives a feedback to the generator for adjustment (red line)

Segmentation problems can be investigated using different neural network architectures. The U-Net, based on the fully convolutional network architecture, has proven to be very suitable for two-dimensional problems. The U-Net can be used in its form in an interplay of Generative Adversarial Networks (GAN). In this work, a conditional generative adversarial network (cGAN) architecture is used for multiclass face analysis in infrared images (Fig. 4).

### U-Net

As a gold standard, the U-Net presented by Ronneberger et al. in 2015 is often used for segmentation problems [10]. The U-Net consist of two major paths; the encoder path (convolutional layers) and the decoder path (deconvolutional layers). The encoder path performs down-sampling and is similar to typical convolutional layers in convolutional neural networks (CNN). The layers are 3 × 3 convolutions. Each deconvolution is followed by a 2 × 2 max pooling layer. The max pooling layer simply samples down the given input in each stage. Through a moving rectangular window, it computes the maximum of each specific window. Afterwards, the output is a smaller output matrix than its input. By batch normalization after each convolutional stage, the output is normalized again to stabilize the network and reduce overfitting. The down sampled feature map, created by the encoder path, is then enlarged again in the decoder path. Due to the symmetrical architecture, the deconvolution layers have the same size as the convolutional layers of the encoder stage. In this way a loss of relevant image information from previous stages is counteracted. Thus, at every decoder step, features from the encoder path with the same dimension are concatenated to the encoded feature. In general, the output dimension is the same as the input. Filter sizes of each layer should be adapted to size of the input image and are defined by a power of 2. An essential advantage of the U-Net is the stable training with a relatively limited data set. However, the U-Net is used here as part of the conditional generative adversarial nets (cGAN) architecture.

### Generative Adversarial Network (GAN)

Generative Adversarial Network (GAN) is a unique architecture of neural networks in which two networks act in opposition to each other and thus learn from each other. GANs were first introduced in 2014 by Ian Goodfellow et al. [11]. The fundamental principle of GANs works as follows: two neural networks work against each other and learn from each other's results. The first network, the generator, receives a random signal as input and tries to generate data that are indistinguishable from the real data. The generated image, together with a part of the training data set, forms the input of the second network, the discriminator. The task of the discriminator is to decide which images come from the training data set and which from the generator. The discriminator learns from its predictions in comparison with the ground truth. Typical fields of application of GAN is, for example, the generation of photo realistic super resolution images (SRGAN) [12]. It is also used in the transformation of black and white images from old photographs or videos into the color space [13]. However, in 2017, Isola et al. presented an extended approach for image-to-image translation with conditional generative adversarial nets (cGAN). This approach was also applied in this work. Whereas GANs learn a generative model of data, conditional GANs (cGANs) learn a conditional generative model [14]. In cGAN architecture, the generator is conditioned on an input image and generates a corresponding output image. The generator is realized by a U-Net. The discriminator is supplied with the output of the generator as well as with the ground truth result image and has to determine whether the result closely matches the original ground truth. That means the discriminator distinguishes between real and fake data and is realized by a convolutional neural network (CNN) which finally distinguishes between 0 and 1. The generator is updated via traditional L1 distance. The L1 distance is calculated by comparing the generated image with the expected output image. By adjusting the loss of the generator model, the generator produces real transformation of the input image. The cGAN architecture was chosen for this multiclass classification instead of the U-Net, because an adaptive loss is learned, which adapts depending on the data. A traditional approach would require a special class-specific weighting and modification of the loss functions. No pretrained weights were used to train the cGAN.

In [14] it is stated that the generator network $G$ tries to minimize the loss against the adversarial discriminator network $D$ which tries to maximize its loss. This results in a final adversarial function for cGANs

1 $$ G^{*} = \arg \min_{G} \max_{D} {\mathcal{L}}_{cGAN} \left( {G,D} \right) + \lambda {\mathcal{L}}_{L1} (G). $$

More information about the network architecture and the derivation of the network loss function, as described in Eq. 1, can be found in [11] and [14]. In this thesis, the existing architecture was taken up and adapted and applied accordingly for NDT problems. The generator was optimized by the Adam [15] optimizer with a learning rate of $$l_{r} = 0.002$$ and an exponential decay rate of $$ \beta = 0.5$$. Deviating from the implementation described in the original paper, stochastic gradient descent (SGD), Adam was chosen as the optimizer for the discriminator network. In this way, a more stable training could be achieved. The learning rate for the discriminator was $$ l_{r} = 0.002$$. We reduced the input image resolution from 320 × 256 pixel to 256 × 256 pixel. This is because of the defined input dimension of the cGAN as well as to speed up training. Furthermore, all images were normalized individually in the range of [-1, 1] by

2 $$ IM_{normalized} = 2\frac{{IM - \min \left( {IM} \right)}}{{\max \left( {IM} \right) - \min \left( {IM} \right)}} - 1 $$

where $$IM$$ denotes the respective two-dimensional IR image to be normalized in the spatial dimensions ($$x,y$$). This step is therefore important to ensure that all of the images have a similar data distribution. As a result, the network is able to converge faster in the learning process. The architecture used is originally optimized for three channel color images. Therefore, the single channel infrared data was stacked into three channels for the training process. Due to the 2D convolution layer used, the tensor size for the training data and the segmentation data can be given as $$ B_{S }\, x \,H \,x \,W \,x\, C $$. The batch size is defined through $$ B_{S }$$. $$H$$ and $$W$$ represents the spatial image dimensions and $$C$$, the channel dimension respectively.

### Data Augmentation

Several forms of data augmentation were performed during the training. The data was randomly rotated in the range 0–90°, zoomed, and also shifted horizontally or vertically. Additionally, elastic deformations on the images were applied. A grid transformation is performed, which calculates an interpolated displacement for each pixel of the input image data. The transformation is applied to the infrared image as well as to the corresponding mask. The elastic deformation approach is described in [10] and [16].

### Training Procedure

The training of the network was carried out on a Linux computer system with Ubuntu 20.04 64-bit. The system used is equipped with a NVIDIA GeForce GTX 1650 graphics processing unit (GPU) with 4 GB of memory. The CPU was a AMD Ryzen™ 7 3750 h with 4 cores with 3.2 GHz each. All neural network models were implemented and tested with TensorFlow 1.13.1 using Python 3.7. With typical CNN architectures, a training termination is usually given when the loss cannot be further minimized. Training GAN networks is more difficult due to the contrary function of generator and discriminator. We found a suitable time for training termination when the generator loss was minimized and the discriminator loss converged at the same time. For the training, the data set of 240 images was divided into 80% training data (192 images) and 20% test data (48 images). The test data was not shown to the network during the training process and is therefore regarded as unknown data. By evaluating test data after each training iteration, we were able to evaluate the performance of the network at this particular stage using intersection over union (IoU) metrics.

### Evaluation Metrics

The results were evaluated using the Intersection over union (IoU) metric. IoU is an evaluation method for determining the accuracy of detection. It compares the segmentation areas of the test images with those output by the trained model. For this purpose, the overlap of the two surfaces is divided by the total area of this surface. The following equation describes the IoU:

3 $$ IoU = \frac{A \cap B}{{A \cup B}} $$

A ∩ B represents the intersection of the predicted and ground truth segmentation mask, while A ∪ B their union. IoU can also be written as follows:

4 $$ IoU = \frac{true\,positive}{{true\,positive\,+\,false\,positive\,+\,false\,negative}}. $$

A prediction result is marked as correctly positive if the IoU is greater than 0.5. In the following, the IoU value for each class of unknown test data was calculated when analyzing the data. In this way, we have a quantitative metric of how accurately each class has been recognized.

## Results and Discussion

### Test Data Results and Quantitative Analysis

**Fig. 5:**
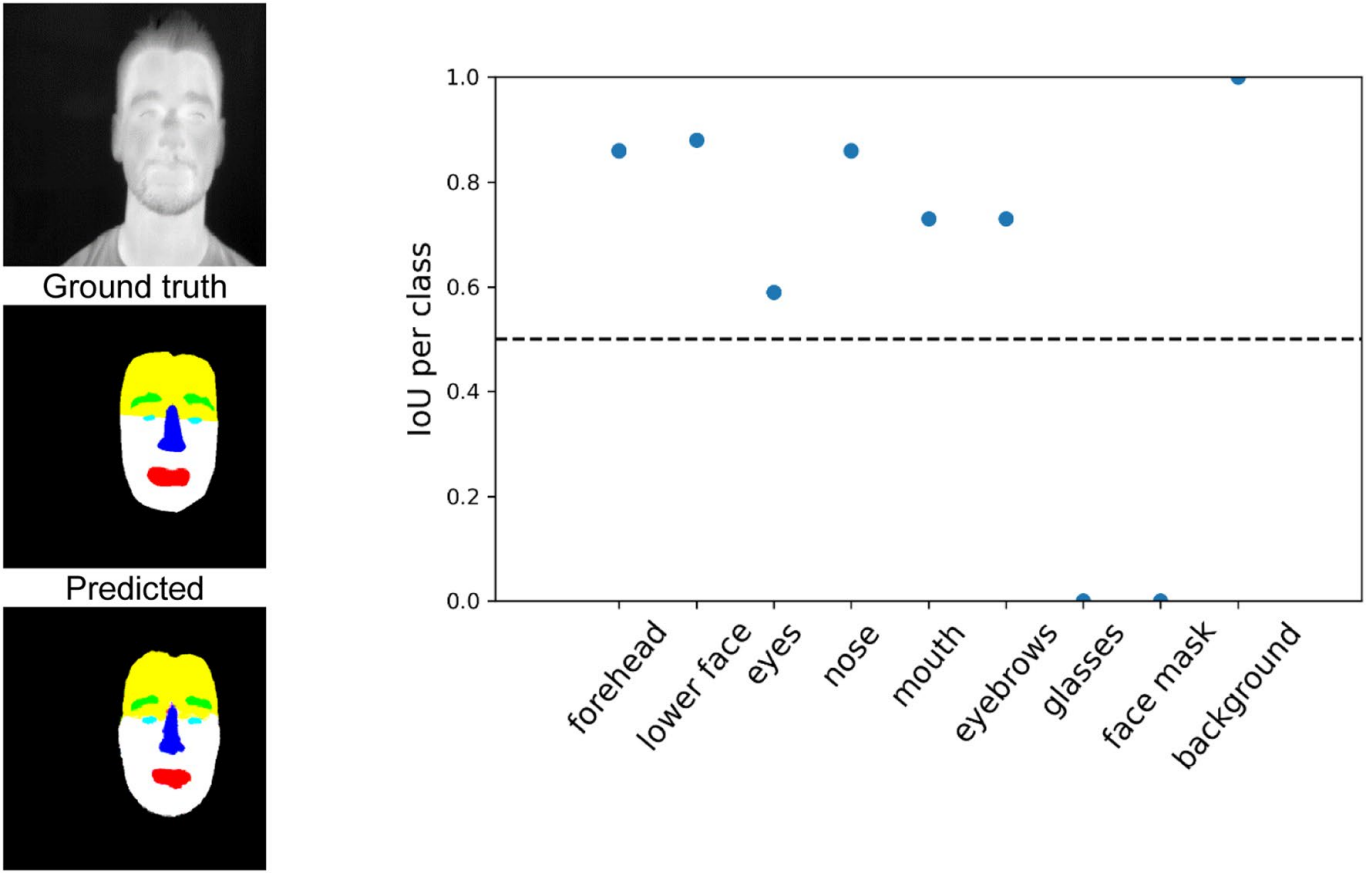
Segmentation results for a participant without glasses or facial mask. For comparison the predicted mask is shown next to the IR image and the ground truth mask. The diagram on the right shows the IoU value for each class. IoU values above the dotted line of 0.5 indicate a good segmentation result

Figure 5 shows some prediction results on the test dataset with the associated multiclass ground truth masks and the cGAN predicted image. The calculated IoU for each class is given. IoU values greater than 0.5 indicates a strong classification result. If the IoU value is 0, the class is not present in the data shown.

**Fig. 6:**
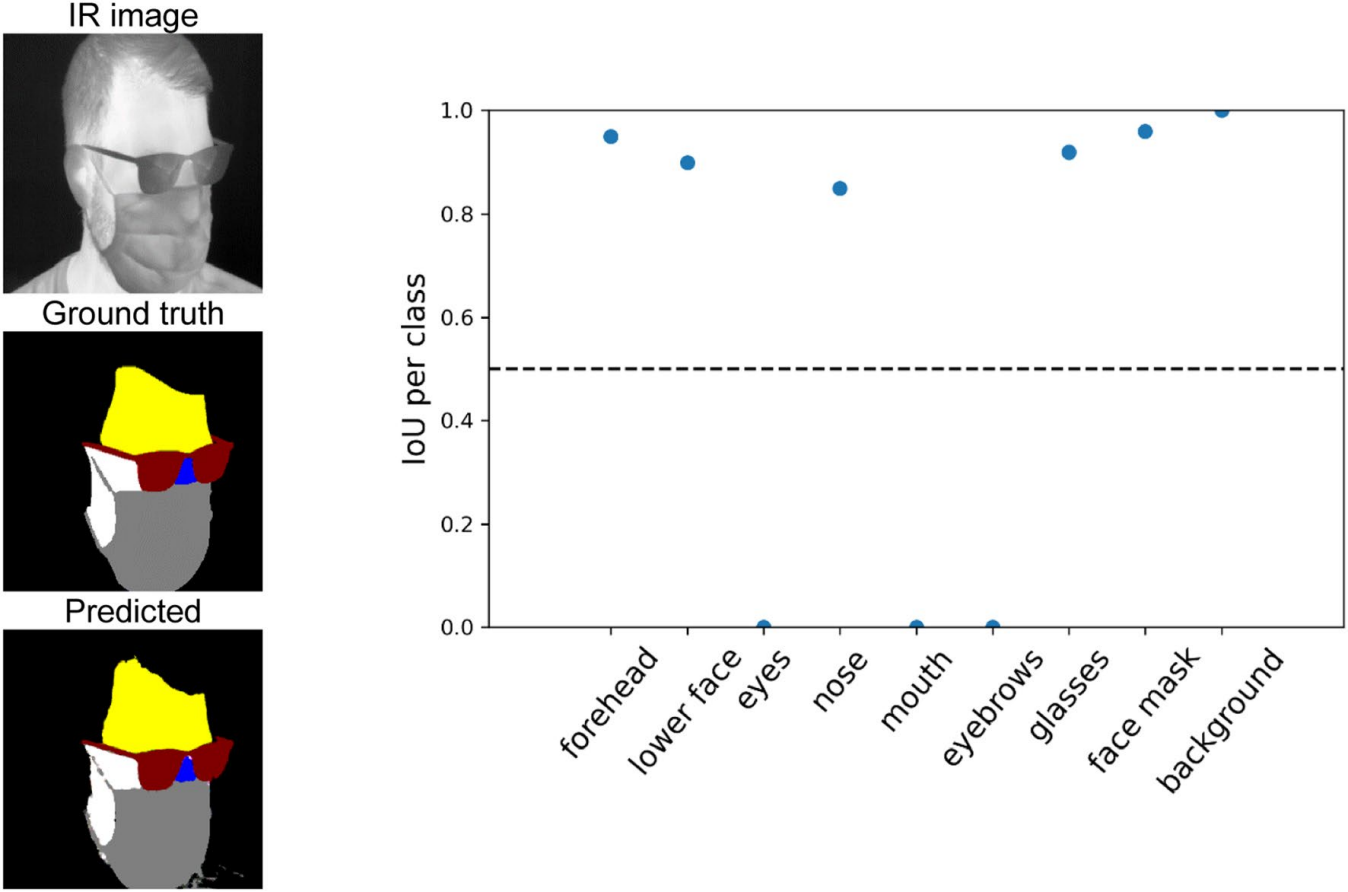
Segmentation results for a participant with glasses and facial mask

From the result shown in Fig. 5, it can be seen that all classes were well identified. Comparing the Ground truth mask with the prediction, it seems that the network has an even more accurate and realistic estimation than the actual Ground truth. It is noticeable that the IoU of the background is close to 1. This indicates that the network is highly capable of distinguishing faces from the environment. Non-existent classes like the glasses or face mask are at an IoU < 0.5, close to 0. Figure 6 shows another result, but with a participant wearing face accessories like glasses and face mask.

The multiclass segmentation mask for the participant with glasses and face mask could be predicted fairly accurately. Non-existent areas such as the mouth, eyes and eyebrows are not shown in the predicted segmentation image. Accordingly, their IoU value is 0. All other detected segments in the predicted mask show a strong IoU > 0.8. This indicates a very good detection rate.

**Fig. 7:**
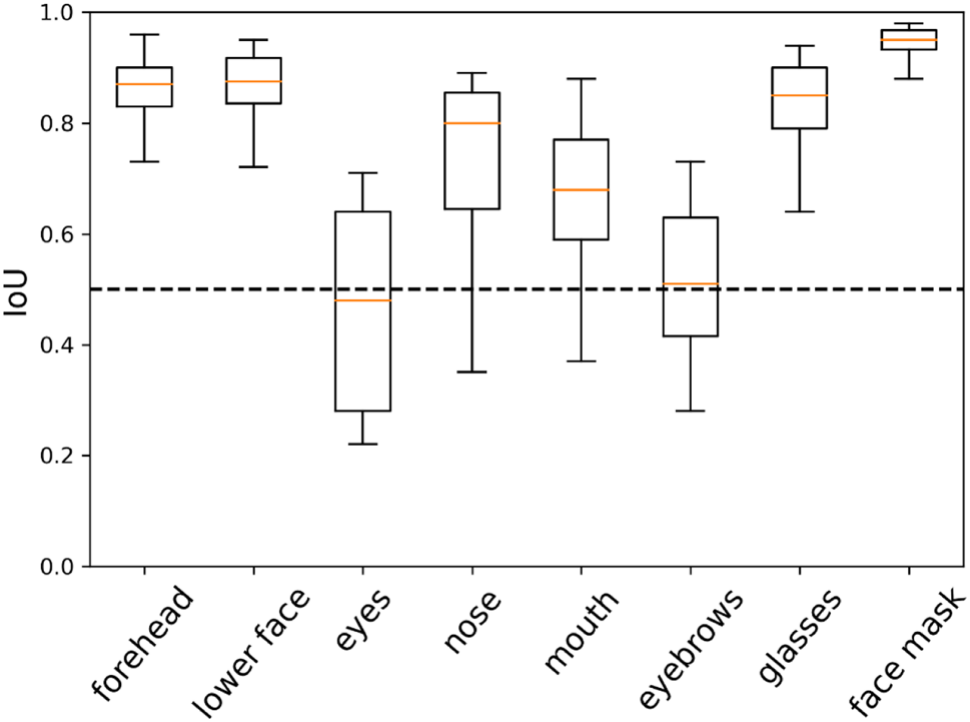
Box-and-whisker plot for the IoU values for segment area among the predicted test dataset. The whiskers indicate the minimum and maximum IoU of the represented class. The mean value is indicated as an orange line. The IoU data spread of each class is represented by the surrounding black box

The performance of the cGAN on the test data set was further evaluated by a box-and-whisker plot, Fig. 7. The represented box-and-whisker plot shows the IoU distribution for each segmentation class of the predicted test dataset. Mean values are indicated as orange lines for each class. The IoU data spread is represented by the surrounding black box, respectively. The whiskers show the minimum and maximum IoU of the different classes. A dotted line at IoU = 0.5 indicates the threshold at which a result is considered to be reasonably predicted.

In the box-and-whisker plot overview of Fig. 7, the mean value of IoU for all classes is above 0.5. An exception is the class "eyes". There, the IoU value is below the threshold value of 0.5 on average. This can be explained by the fact that when the masks were created manually, the segmentation mask for the eyes are were difficult to create. Therefore, an error has occurred when labeling the data. There are also outliers for the classes "nose", "mouth", and “eyebrows” below the threshold value of 0.5. These could be incorrectly classified segmented areas. In general, it can be said that classes, which take up a larger image area, were detected better and with more stability. Overall, a quite solid result can be presented with a high level of accuracy. The eyes with the smallest image area consequently show the worst results here as well.

### Live Infrared Sequence Analysis

**Fig. 8:**
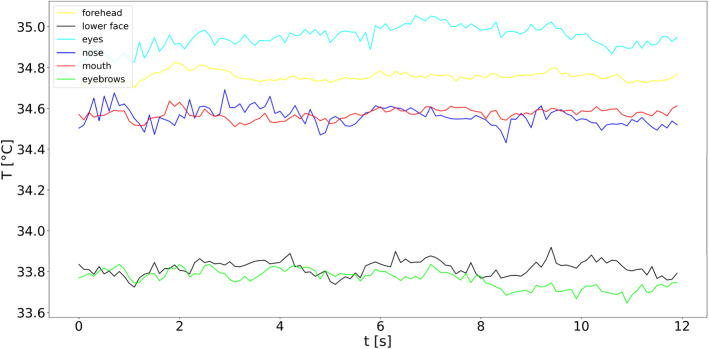
Time temperature signal curves for the individual segmentation areas of the face. The legend in the upper left corner shows the corresponding class name for the coloration of single signals

In the following section, a live sequence analysis for one participant is shown with the corresponding temperature extraction for each segmentation class. For the live image analysis, an additional data set of 12 s was acquired. For the time sequence recording, the participant did not wear glasses and mask. Figure 8 shows the time temperature signal curves for the individual segmentation areas of the face. The temperature is indicated in °C. The selected color code for the individual signals corresponds to the colors selected of the created segmentation masks. Note that a different color was selected for the class "lower half of the face", in this case black, because a white curve on a white background does not provide visible contrast.

All facial areas in the dataset could be detected and are displayed as stable over the complete recorded time. For the visualization of the time curves, the average values of the individual segmentation class were calculated for each frame. It is clearly visible that the different areas of the face have different temperatures. Furthermore, it is obvious that the eyes and the forehead area have a higher temperature than the surrounding areas. The major variation of the temperature signal of the class eyes compared to the other classes in Fig. 8 can be explained by inaccurate created ground truth masks and the resulting misclassifications. This thesis is also supported by the box-and-whisker plot for the class eyes, which IoU mean is < 0.5 in Fig. 7. Areas such as the eyebrows and the lower half of the face are colder than the other classes in the signal curves shown. Especially in the lower half of the face, no distinction was made between skin and facial hair, which means that a beard can have an influence on the result. Through the temperature characteristics it is clear that a temperature distinction can be made between the individual facial areas. In terms of e.g. disease or infection detection, a simple range of interest around the entire facial area is inadequate for temperature analysis. The data are evaluated in a few microseconds by an appropriate computer hardware. This enables a stable live image analysis.

## Conclusion and Further Work

**Fig. 9:**
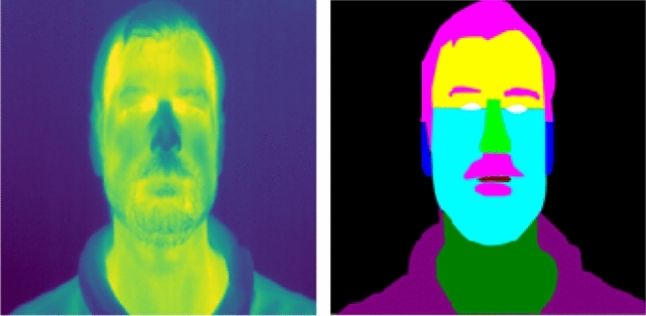
Possible extension of the multiclass segmentation mask. Shown here are classes for: clothing, neck, lower face, ears, mouth, nose, facial hair, eyes, forehead

In this work we have adapted an approach for the end-to-end face segmentation in the thermal infrared and applied it successfully. With this approach, it could be shown that a pixel accurate automatic thermal analysis is possible. This allows each previously defined area of an IR image to be examined individually. As a demonstrated application, in the context of the Covid-19 pandemic, the approach to face analysis and possible temperature detection was shown. In order to be able to use the presented approach in the medical environment, the results must be analyzed by a medical expert. This would allow for a precise definition of which temperatures on the skin surface of the different facial areas are critical from a medical point of view and could give an indication of a possible disease or infection. This was not done in the context of this work. Furthermore, the database created and the stability of the network can be improved by a larger group of subjects, but in the context of the Covid-19 pandemic situation it was difficult to motivate subjects in compliance with the current restrictions. In order to further demonstrate the approach and to evaluate the functionality, a sufficiently larger database could be produced. However, in order to be able to use the method permanently in a real environment (e.g. train stations, airports, universities and shopping malls), the database would have to be enlarged by a larger group of participants with different ages and gender. This challenge is to be considered in a currently starting R&D project which applies various in-field-test situations for data generation. In particular, it would be important that people of different ages, skin colors, and origins are trained to the network. Furthermore, the work could also be extended by implementing emissivity correction for every predicted face class. For this purpose, the individual classes could be assigned to different emissivities e.g. for glasses, clothes or hair. Additionally, the presented work could be extended by a larger group of sub facial classes, see Fig. 9.

The predefined segmentation mask areas could e.g. be divided even more precisely: the lower half of the face, can be divided into subclasses such as the cheek area or chin. Furthermore, two classes can be defined for the nostrils. In this way, a possible respiratory output with respiratory rate could be segmented from the IR image. The presented procedure represents the progress in the transfer of existing neural approaches in the field of NDT. This approach can also be used for multi defect detection and multi class classification in material analysis in the scope of NDT4.0. Further work will investigate the transferability of the approach to other NDT problems.
